# Gut Microbiota and Gastrointestinal Polyps: Unveiling the Causal Connection

**DOI:** 10.5152/tjg.2025.0201254

**Published:** 2024-05-02

**Authors:** Tarkan Karakan

**Affiliations:** 1 Department of Gastroenterology, Gazi University Faculty of Medicine, Ankara, Türkiye

The gastrointestinal (GI) tract hosts a complex microbial community that profoundly influences human health, affecting immune modulation, nutrient metabolism, and gut homeostasis. Recent insights from genetic epidemiology have now elucidated a causal role for gut microbiota in various gastrointestinal diseases, including polyps, which have potential for malignant transformation. The study by authors published in this issue of the Turkish Journal of Gastroenterology elegantly utilizes Mendelian randomization (MR) analysis to explore the causal relationship between gut microbiota and gastrointestinal polyps in different segments of the GI tract.

This innovative MR approach, leveraging genome-wide association study (GWAS) data, circumvents limitations typically encountered in observational studies, such as confounding factors and reverse causation.^1-3^ Utilizing the extensive MiBioGen database and gastrointestinal polyp data from the MRC-IEU Consortium, the authors have established significant associations between certain bacterial taxa and polyp formation. Notably, *LachnospiraceaeUCG004, ErysipelotrichaceaeUCG003, and Veillonella* were identified as risk factors for colon polyps, whereas *Dorea and Clostridium innocuum* group demonstrated protective roles. Similarly, *Allisonella* emerged as a risk factor for rectal polyps, whereas *ChristensenellaceaeR.7group, Intestinimonas, and Parasutterella* were protective. For gastric and duodenal polyps, *LachnospiraceaeFCS020* group, *Intestinibacter, RuminococcaceaeUCG003, and Parasutterella* were recognized as risk factors.

Previous research has demonstrated a relationship between intestinal microbiota and gastrointestinal disorders, such as irritable bowel syndrome, inflammatory bowel disease, and colorectal cancer (CRC).^4,5^ Additionally, gut microbiota has been implicated as an important factor influencing the progression from colorectal adenomatous polyps toward malignant transformation into CRC.^6^

A recent investigation highlighted that higher levels of *Fusobacterium mortiferum* may be associated with colorectal polyp formation.^7^ Another research identified the *Prevotella* enterotype as distinctly prevalent among individuals with colorectal adenomas, implying its potential role in the pathology of this condition.^8^ Additionally, distinct variations in the gut microbiome have been observed when comparing healthy individuals to patients diagnosed with serrated polyps. Thus, these unique microbiota changes observed in premalignant colorectal polyps might serve as potential biomarkers for early detection of colorectal cancer.

Nevertheless, this study’s limitations must be acknowledged. The genetic datasets used were primarily of European descent, thus limiting the generalizability of results across diverse ethnic populations.^9^ Further research is imperative to confirm these findings universally and to elucidate the exact mechanisms through which gut microbiota influence polyp formation.

A study published in 2024 employed Mendelian randomization (MR) analysis to investigate the causal links between gut microbiota and various types of polyps, including nasal, gallbladder, colon, and gastric polyps.^3^ The findings revealed significant associations between specific bacterial taxa and polyp development. Notably, certain bacteria were identified as risk factors for colon polyps, while others demonstrated protective roles.

In the context of colorectal cancer (CRC), a 2025 study highlighted the significant impact of intestinal microbiota on the progression from colorectal adenomatous polyps to CRC. The research identified specific bacterial phyla associated with colorectal adenomas, underscoring the potential of microbiota-based biomarkers for early detection and targeted therapies.^10^

Furthermore, a comprehensive review in 2025 discussed the role of gut microbiota in CRC, emphasizing the mechanistic interplay between microbiota composition, the intestinal barrier, and the immune system. The review suggested that probiotics and fecal microbiota transplantation offer potential strategies for CRC prevention and treatment by restoring microbial balance and enhancing anti-cancer immune responses.^11^

Of note, gut microbiota is not only composed of bacteria. There are other members of this huge kingdom, such as fungi, viruses (bacteriophages) and archea. Very few studies investigated fungal microbiome in CRC and polyps.^12,13^ The top ten dominant fungal taxa in patients with colorectal polyps are: *Mortierella echinula* (19.70%), *Saitozyma podzolica* (9.27%), *Purpureocillium lilacinum* (5.82%), *Aureobasidiaceae* (family level) (5.77%), Trichoderma virens (5.41%), Apiotrichum sporotrichoid (4.32%), Paraphaeosphaeria (genus level) (3.00%), *Trichoderma* (genus level) (2.96%), *Aspergillus* (genus level) (2.94%), and *Xylomyces* (genus level) (2.77%). It was suggested that *Mortierella echinula, Aureobasidiaceae* (family level) and *Trichoderma virens* have a potential association with both CRC and polyp patients, as they were abundantly presented in both clusters.^12^ Virome and archea are not thoroughly investigated in colorectal polyps.

Last but not least, the metabolic functions of gut microorganisms are the most important factor in the pathogenesis of polyp formation. Recent studies highlight the potential of metabolites that are produced by certain bacterial groups.^14,15^

In conclusion, the study by authors published in this issue of the Turkish Journal of Gastroenterology [May 2025 issue] significantly advances our understanding of the microbiota-polyp interaction. It not only identifies specific bacterial taxa involved in polyp formation but also suggests practical implications for screening, prevention, and treatment strategies tailored around microbiota modulation. Future research should prioritize clinical trials and mechanistic studies to validate these intriguing associations and explore therapeutic interventions aimed at restoring microbiota balance, thereby reducing gastrointestinal polyp risk and its associated malignancy potential.

## Data Availability

The data that support the findings of this study are available on request from the corresponding author.
